# Neurodevelopmental outcome for offspring of women treated for antenatal depression: a systematic review

**DOI:** 10.1007/s00737-014-0457-0

**Published:** 2014-09-12

**Authors:** Giovanni Previti, Susan Pawlby, Sahmina Chowdhury, Eugenio Aguglia, Carmine M. Pariante

**Affiliations:** 1 Department of Psychological Medicine, Institute of Psychiatry, King’s College London, Room 2-055, The James Black Centre, 125 Coldharbour Lane, London, SE5 9NU UK; 2 Department of Clinical and Molecular Biomedicine, Section of Psychiatry, University of Catania, School of Medicine, Catania, Italy

**Keywords:** Antenatal depression, Neurodevelopment, Depression treatment, Perinatal, Pregnancy outcomes

## Abstract

The aim of this systematic review is to appraise existing literature on the effects of treatments for antenatal depression on the neurodevelopment outcomes of the offspring. We conducted a systematic review of the literature to identify studies on different kinds of treatments for antenatal depression (antidepressants and alternative therapies) and their effects on infants’ neurodevelopment. After reading the title, abstract, or full text and applying exclusion criteria, a total of 22 papers were selected. Nineteen papers studied the effects of antidepressant drugs, one on docosahexanoic acid (DHA) (fish oil capsules) and two on massage therapy; however, no studies used a randomized controlled design, and in most studies, the control group comprise healthy women not exposed to depression. Comparisons between newborns exposed to antidepressants in utero with those not exposed showed significant differences in a wide range of neurobehavioral outcomes, although in many cases, these symptoms were transient. Two studies found a slight delay in psychomotor development, and one study found a delay in mental development. Alternative therapies may have some benefits on neurodevelopmental outcomes. Our review suggests that antidepressant treatment may be associated with some neurodevelopmental changes, but we cannot exclude that some of these effects may be due to depression per se.

## Introduction

Pregnancy is a sensitive period for a woman’s health, both physical and psychological. It is the time when a new life forms in her body and many hormonal variations take place which can affect mood (Ahokas et al. 2005). In the last two decades, research into the well-being of women around childbirth has increasingly focused on depression in the antenatal rather than in the postnatal period. Studies from different countries have estimated the prevalence of antenatal depression to range from 6 to 38 %. Different studies have found that compared to non-pregnant women, pregnant women at any gestational age have an increased chance of suffering from a mood disorder such as major or minor depression (Dietz et al. 2007; Marchesi et al. 2009; Marcus 2009). Antenatal depression has often been found to be more frequent than postnatal depression, in some cases the estimate being twice that of depression following birth (Field 2011). Moreover, women suffering from antenatal depression are at high risk of developing depression in the postnatal period (Leigh and Milgrom 2008; Marino et al. 2012). According to a number of studies, the highest risk periods for developing a mood disorder are the first and last trimester of pregnancy (Gavin et al. 2005; Field 2011; Marchesi et al. 2009).

Numerous studies and a recent meta-analysis have shown that depression throughout pregnancy may lead to complications (preeclampsia, spontaneous miscarriage) and poor outcomes for the offspring, such a slow intrauterine growth, placental abnormalities, low birth weight, preterm birth, and frequent admission to the neonatal intensive care unit (Bonari et al. 2004; Deave et al. 2008; Field et al. 2006; Grote et al. 2010). Neonates of depressed mothers perform poorly on many clusters of the Brazelton Neonatal Behavioral Assessment Scale (NBAS) such as orientation, reflex, excitability, and withdrawal; they are also more aroused and less attentive (Hernandez-Reif et al. 2006; Hollins 2007; Lundy et al. 1999a). Deave et al. (2008) investigated the long-term effects of perinatal depression on child development, and they found that some of the effects on child development previously believed to be associated with postnatal depression were instead associated with depressive symptoms during pregnancy.

Taking these findings into account, it is important to identify and treat all pregnant women who experience symptoms of low mood or severe depression in order to reduce adverse child outcomes. It is not easy for clinicians and mothers to make decisions about the type of therapy that is appropriate for each individual pregnant woman. Many factors such as the severity of the mother’s disease, the consequences of her illness on the developing fetus, and the long-term outcome for both mother and baby need to be considered. Guidelines have been developed to help clinicians decide with their patients the best form of treatment, giving them adequate information to give informed consent to the therapy. The National Institute for Health and Clinical Excellence clinical guideline underlines that drugs should be prescribed cautiously for women who are planning a pregnancy and for those who are pregnant or breastfeeding. They suggest different therapies based on the severity of disease: subthreshold, mild, moderate, severe, and treatment-resistant depression (NICE clinical guideline 45Antenatal and postnatal mental health Clinical management and service guidanceIssued: February 2007). Wisner et al. (2000) introduced five domains: intrauterine fetal death, physical malformations, growth impairment, behavioral teratogenicity, and neonatal toxicity in her decision-making model to ensure that patients and psychiatrists deal with all the critical risk-benefit aspects. Koren et al. (Koren and Nordeng 2012) in his recent review also emphasized that very little has been done to integrate the potential risks, if they exist, and to allow an evidence-based benefit-risk ratio. Good algorithms to evaluate and manage the risk of exposure to depression or antidepressant were made by the American Psychiatric Association in collaboration with the American College of Obstetricians and Gynecologists (Yonkers et al. 2009).

Despite these documents helping clinicians and patients make appropriate decisions, pregnant women were found to be diffident toward taking antidepressant drugs, with a high percentage of women discontinuing their use, compared to the use of other therapies such as the counseling service provided by the Motherisk Program (Bonari et al. 2005). This reluctance by pregnant women to take antidepressant drugs should lead clinicians to discuss with their patients the use of psychological interventions or alternative forms of treatments such as light therapy, massage therapy, or omega-3 fatty acid supplementation (Dennis et al. 2007; Dennis and Allen 2010). Currently, the most studied category of treatments is antidepressant use and its effects on the mothers and babies. There is some evidence to show that therapies other than drugs are effective forms of treatment for the mother’s mental illness, but to our knowledge, there are no recent systematic reports on the their impact of antidepressant treatment on the newborn focusing on neurodevelopment (Chaudron 2013; Gentile and Galbally 2011; Lattimore et al. 2005), rather than poor neonatal adaptation syndrome or similar reaction at births (Grigoriadis et al. 2013). The aim of this study is to review existing literature on the neurodevelopmental outcome of newborns born to mothers who were treated for antenatal depression with drugs or alternative therapies. Alternative therapies could have a number of beneficial effects on the offspring through direct nutritional effects or by improving symptoms and well-being in the mothers, but, especially considering their widespread use, it is important to identify any potential negative consequences.

## Materials and methods

The words “antenatal or postnatal or antepartum or postpartum or peripartum or prepartum or perinatal or pregnancy or neonatal” and “depression” and “treatment or NBAS or Bayley or neurodevelopment or baby outcome” were searched from 1950 to May 2013 in the PubMed, The Cochrane Library, Scopus, Embase, OvidSP, PsychInfo, and ISI Web of Knowledge. The resultant papers were cross-referenced for other relevant studies not identified in the initial research. An extensive manual research of literature was also done by checking pertinent journal and authors. The only studies selected were ones that assessed pregnant women with depression who were treated with drugs or alternative therapies, and where their newborns were measured specifically by using standardized tests of neurodevelopmental outcomes administered by trained staff. Other papers where symptoms are described based on questionnaire or clinical notes were not included. The tests used for neurobehavioral assessment of the infant were NBAS (Brazelton 1973) and NICU Network Neurobehavioral Scale (NNNS) (Lester and Tronick 2004). The same methodology was used for the selection of studies that assessed neurodevelopment and long-term effects on infants. The tests used were the Bayley Scales of Infant Development (BSID) second and third editions (Bayley 1993, 2005), the Wechsler Preschool and Primary Scale of Intelligence third edition (WPPSI) (Wechsler 2002), the Wechsler Adult Intelligence Scale (Wechsler 1981, 1999), the BOEL test (Junker et al. 1982), the McCarthy Scale (McCarthy 1972), and the Reynell Developmental Language Scale (Reynell 1985).

Including clinical trial, meta-analysis, randomized controlled trial, review, and systematic reviews, we found 2,332 papers in 8 search engines. We excluded papers not written in English or based on animal models, and after reading the title, abstract, or full article, only 22 studies met inclusion criteria (Table 1).

**Table 1:** Studies that met the inclusion criteria

Search engine	Papers	New selected papers
PubMed	546	17
The Cochrane Library	35	0
Scopus	485	1
Embase	513	0
OvidSP	375	0
PsychInfo	160	0
ISI Web of Knowledge	218	0
Added from references	4	4

The majority of the literature focused on the birth outcomes (birth weight, length of gestation, Apgar score) for the infants following treatment of the mother’s depression. To our knowledge, there are only a very few studies that examined short- and long-term neurodevelopmental outcomes for the baby. This is true especially for therapies other than drugs. In 19 papers out of the 22 selected, mothers were treated with selective serotonin reuptake inhibitors (SSRI) as citalopram, fluoxetine, sertraline, paroxetine, fluvoxamine, and escitalopram, or serotonin–norepinephrine reuptake inhibitor (SNRI) as venlafaxine. We found only one paper that analyzed the effects of docosahexanoic acid (DHA)-rich fish oil capsules and two papers that explored massage therapy. Only the studies on alternative therapies were randomized controlled trials with a placebo group of depressed mothers. All the other studies were prospective cohort studies.

## Results

The selected papers were arranged in ascending order according to the age of the babies at the time of assessment, from birth until 86 months old. The following sections evaluate short-term effects (from birth to 8 weeks) (Table 2) and long-term effects (from 2 to 86 months) (Table 3) on offspring neurodevelopmental outcomes of different treatments for antenatal maternal depression.

**Table 2:** Studies on short-term effects

Author, kind of study, scale used to assess depression, and severity	Number of participants	Number of babies assessed	Age of the baby at assessment	Timing of mother exposure	Treatment used	NBAS results	Finding
Ferreira et al. 2007, Canada Retrospective cohort study Scale used and severity not specified.	76 exposed on SSRI or venlafaxine; 90 control	79 exposed to SSRI or venlafaxine; 91 control	At birth	Third trimester	SSRI (paroxetine, fluoxetine, sertraline, citalopram, fluvoxamine) venlafaxine.	All neonatal behavioral signs (tremors, shaking, agitation, spasms, hypertonia or hypotonia, apnea, tachypnea, bradycardia, indrawing, irritability, and sleep disturbances): group 1 = 59 (77.6) group 2 = 37 (41.1) (*p* < 0.001)	Significant differences between newborns exposed and control for NBAS, prematurity, and birth weight. Neonatal behavioral signs were frequent in exposed newborns, but symptoms were transient and self-limited. Premature infants could be more susceptible to the effects of SSRI and venlafaxine.
Zeskind and Stephens 2004, USA Prospective cohort study Review of medical records Severity not specified.	17 exposed to SSRI; 17 control	17 exposed to SSRI; 17 control	At birth	All pregnancy	SSRI (paroxetine, fluoxetine, sertraline, citalopram) and bupropion	Significant differences between groups in a wide range of neurobehavioral outcomes (tremulousness, behavioral states, active sleep)	Significant differences between newborns exposed and control for NBAS and prematurity. Results provide the first systematic evidence that women who use SSRIs during pregnancy have healthy, full-birth-weight newborn infants who show disruptions in a wide range of neurobehavioral outcomes.
Rampono et al., 2009 USA Prospective observational study Edinburgh Postnatal Depression Scale (EPDS) >11	38 exposed to antidepressant (27 SSRI, 11 SNRI), 18 control	38 exposed to antidepressant (27 SSRI, 11 SNRI), 18 control	Between 1 and 6 days	Any time throughout pregnancy, trimester is not specified	SSRI (escitalopram, paroxetine, fluoxetine, sertraline, citalopram, fluvoxamine) venlafaxine.	Significant differences in mean NBAS scores between cases and controls for the habituation, social-interactive, motor, and autonomic clusters were found (*p* < 0.05).	Significant differences between newborns exposed and control in same NBAS scores in the early perinatal period but these were self-limiting and similar for both SSRIs and the SNRI venlafaxine.
Suri et al., 2011 USA Prospective, naturalistic, blinded study SCID-I for DSM-IV, Hamilton Depression Rating Scale (HDRS) Maximum HDRS score across pregnancy: 18.5/16.4/10.7	33 exposed to antidepressant, 16 with a history of MDD not treated during pregnancy, 15 control	31 exposed to antidepressant, 14 whose mothers had a history of MDD not treated during pregnancy, 14 control	First visit: within 1 week; 2nd visit between 6 and 8 weeks	All women took AD for the 2nd and 3rd trimester, majority for all trimester.	Antidepressant (not specified)	Summary scores for the 7 clusters were not significantly different among groups at either visit 1 or visit 2. Some significant differences were noted on rapidity of buildup item at visit 1, inanimate auditory and defense items at visit 2 but after Bonferroni correction none significant differences.	No significant differences in summary scores of the NBAS among the three study groups. Use of antidepressants in pregnancy was not associated with significant neurobehavioral effects in infants.
Field et al., 2009, USA Randomized clinical trial SCID-I for DSM-IV, Center for Epidemiological Studies-Depression (CES-D): 23.7/20.3	88 massage group, 61 untreated depressed (standard treatment)	88 massage group, 61 untreated depressed (standard treatment)	At birth	Second and third trimester (20–32 weeks)	12 weeks massage therapy (twice per week)	The massage group neonates received significant higher scores on habituation, orientation, motor, and depression scores. In addition, they had lower cortisol levels.	Significant differences between newborns of depressed mothers treated with massage therapy and untreated for NBAS, prematurity, low birth weight, and they had lower cortisol levels. The group of treated women reduced depression scores by the end of the therapy period and cortisol levels during the postpartum period.
Field et al., 2004, USA Randomized clinical trial Profile of Mood States Scale (POMS), CES-D: 24.9/26.2/28.3/6.5	84 depressed women divided in three groups: 28, massage therapy; 28, muscle relaxation; 28, standard prenatal care; 28 control	84 depressed women divided in three groups: 28, massage therapy; 28, muscle relaxation; 28, standard prenatal care; 28 control	Within a few days after birth	Second and third trimester	16 weeks massage therapy (twice per week)	Significant difference in habituation, range of state, state, autonomic stability, withdrawal, depressed, motor maturity.	Significant better NBAS score for the mother’s baby treated with massage therapy.
Smith et al. 2013 USA Prospective study Composite International Diagnostic Interview v2.1 (CIDI), EPDS: 7.67/5.18	6 exposed to SSRI, 61 control	5 exposed to SSRI, 41 control	24 h age (±8 h)	At least more than 1 month during the third trimester	SSRI (fluoxetine, citalopram, sertraline)	Significant difference in motor cluster scores: 25.2/28.9 (S). No other significant differences.	Significant differences between newborns exposed to SSRI and control mothers, the former had poorer motor development at NBAS, lower 5-min APGAR scores, and shorter mean gestational age as compared to unexposed infants. No significant differences in infant sleep state and number of startless and tremulousness.
Salisbury et al. 2011 USA Prospective, naturalistic cohort study SCID-I for DSM-IV, RSD: 3.8/13.5/12	76 control, 7 depressed untreated, 46 depressed treated	56 control, 20 depressed untreated, 36 depressed treated	Between 1 and 21 days	At least four consecutive weeks during the second and/or third trimesters of this pregnancy.	SSRI	NICU Network Neurobehavioral Scale (NNNS); MDD group infants had lower attention scores compared to CON group infants and MDD + SRI group infants (S). MDD + SRI group infants had lower quality of movement scores and more CNS stress signs than infants in the CON groups (S). Hypertonicity and arousal were significant in the overall model.	Newborns exposed to maternal depression and SSRI treatment during pregnancy had higher risks of different neurobehavioral profiles than control group in the first month of life.

**Table 3:** Studies on long-term effects

Author, kind of study, scale used to assess depression and severity	Number of participants	Number of babies assessed	Age of the baby at assessment	Timing of mother exposure	Treatment used	Test used and results	Finding
Oberlander et al. 2004, Canada Prospective cohort study VScale used and severity not specified.	28 exposed to SSRI; 28 exposed to SSRI + BDZ 23 control	28 exposed to SSRI; 18 exposed to SSRI + BDZ 23 control	At birth, at 2 and 8 months	The majority of women exposed from the first trimester and continued throughout their pregnancy	SSRI (paroxetine, fluoxetine, sertraline)/clonazepam	Bayley: At 2 months: Mental Developmental Index (MDI) = 97/94/96.7 (NS); Psychomotor Developmental Index (PDI) = 104.8/102.9/102.6 (NS). At 8 months: MDI = 100.7/97.2/99.4 (NS); PDI = 91.5/93.1/97.0 at 8 months (NS).	No significant differences in Bayley scores. Transient neonatal symptoms were found at birth in infants after single-agent prenatal exposure to SSRIs and when paroxetine was combined with clonazepam.
Morison et al. 2001, Canada Prospective cohort study Scale used and severity not specified.	18 exposed to SSRI; 20 control	Not reported	Between 2 and 8 months	Not reported	SSRI (paroxetine, fluoxetine, sertraline)	Bayley (no results showed, only abstract)	No difference on BSID total score between exposed and control group.
Reebye et al. 2002, Canada Prospective cohort study Hamilton Depression Rating Scale (HDRS) Scores not reported	24 exposed to SSRI; 14 exposed to SSRI + clonazepam; 23 control	24 exposed to SSRI; 14 exposed to SSRI + clonazepam; 3 control	2 months	Not reported	SSRI (paroxetine, fluoxetine, sertraline)/clonazepam	Bayley: *I* = 98/93/96 (NS), PDI = 106/102/101 (NS).	No significant difference in Bayley scores, birth weight and gestational age.
Mortensen et al. 2003, Denmark Follow-up study Scale used and severity not specified.	435 women on psychotropic drugs (50 on antidepressant), 1,304 control	340 exposed to drugs, 755 control	Between 7 and 10 months	During all pregnancy, trimester isn’t specified	BDZ, AD, anti-epileptic drugs, neuroleptic drugs	16 % of baby exposed on antidepressant had abnormal Boel test, 4 % of abnormal test in the control group. Adjusted OR 5.9 (1.1–31.0)	Higher percent of abnormal Boel test among those who were exposed to antidepressants compared to the non-exposed group.
Hanley et al. 2013, Canada Prospective study Edinburgh Postnatal Depression Scale (EPDS): 6.2/5.0 HRDS: 10.6/7.2	31 SSRI, 52 control	31 SSRI, 52 control	10 months	All pregnancy	SSRI (paroxetine, fluoxetine, sertraline, citalopram) and venlafaxine	Bayley: Cognitive 10.9/11.5 (NS); Communication: Receptive 10.2/10.2 (NS) Expressive 9.8/9.9 (NS) Motor: Fine 11.2/11.3 (NS) ross 9.5/8.3 (S) Social–emotional 9.6/8.5 (S) Adaptive behavior 73.3/68.4 (S)	Infants prenatally exposed to SRIs score significantly lower on the gross motor, social–emotional
Makrides et al. 2010, Australia Randomized clinical trial EPDS > 12	2,399 depressed women: 1,197 treated with DHA supplement; 1,202 treated with control supplement	351exposed to DHA supplement; 375exposed to control supplement	18 months	Second trimester	Docosahexaenoic acid-rich fish oil capsules (providing 800 mg/day of DHA)	Bayley: cognitive standardized score = 101.8/101.75 (NS), language standardized score = 96.47/97.94 (NS), motor standardized score = 102.63/102.57 (NS), social-emotional standardized score = 106.32/107.27 (NS), adaptive behavior standardized score = 99.17/100.75 (NS).	No significant differences in cognitive, motor, social-emotional, adaptive behavior, and language scores between groups in Bayley scores between newborns exposed to DHA-rich fish oil capsules and vegetable oil capsules during pregnancy. The use of DHA-rich fish oil capsules did not result in lower levels of postpartum depression in mothers or improved cognitive and language development in their offspring during early childhood.
Casper et al. 2011, USA Prospective cohort study SCID-I for DSM IV BDI: 25.3/25.6/19.6	55 exposed to SSRI: 14 on 1st trimester, 18 2nd and 3rd, 23 all pregnancy)	55 exposed to SSRI: 14 on 1st trimester, 18 2nd and 3rd, 23 all pregnancy)	Between 12 and 40 months	1st, 2nd, and 3rd trimester	SSRI (paroxetine, fluoxetine, sertraline, citalopram)	Bayley: MDI = no association with duration of SSRI exposure. PDI = significant negative correlations with length of exposure. Behavior Rating Scale (BRS) = significant negative correlation with the duration of exposure.	Significant negative correlation between the length of exposure and psychomotor development and Behavior rating scale. No significant correlation with mental development. Timing of exposure to SSRIs during susceptible periods of fetal development and variations in the severity of maternal depression may have contributed to the associations.
Nulman and Koren 1996, Canada Prospective cohort study Scale used and severity not specified.	55 exposed on fluoxetine, *N*Control group not specified	55 exposed to fluoxetine (37 on 1st trimester, 18 throughout pregnancy)	Between 18 and 30 months	37 exposed during 1st trimester, 18 during all pregnancy	SSRI (fluoxetine)	Bayley: MDI = 117/115 (NS), McCarthy: General Cognitive Index (GCI) = 114/114 (NS)	No significant differences in Bayley mental development index and in McCarthy scores between newborns exposed to fluoxetine and control. A significant correlation was found between maternal and child’s IQ.
Nulman et al. 1997, Canada Prospective cohort study Global assessment scale: 62/60/-Center for Epidemiological Studies-Depression (CES-D): 33/35/–	80 exposed to TCA; 55 exposed to fluoxetine; 4 control	80 exposed to TCA; 55 exposed to fluoxetine; 84 control	16–86 months	TCA: 40 exposed during the 1st trimester, 36 all pregnancy, 2 1st and 2nd trimesters, 2 1stand 3rd trimesters. SSRI: 37 exposed during 1st trimester, 18 during all pregnancy	TCA, fluoxetine	Bayley: MDI = 118/117/115 (NS) McCarthy: GCI = 117/114/114 (NS) Reynell Developmental Language Scales: Verbal Comprehension Scale = 1.3/1.2/1.1 (NS) Expressive Language Scale = 0.3/–0.2/0.1 (NS)	No significant differences in cognitive, language, and behavioral development among the children who were exposed to antidepressant drugs in utero and those who were not. In utero exposure to either TCA or fluoxetine does not adversely affect the neurodevelopment of pre-school children.
Nulman et al. 2002, Canada Prospective study CES-D: 39.9/28.0/10.7	40 exposed to fluoxetine; 46 exposed to TCA; 36 control	40 exposed to fluoxetine; 46 exposed to TCA; 36 control	At birth, between 15 and 71 months	All pregnancy	TCA, fluoxetine	Bayley: MDI = 104.4/110.9/104.1 (NS), PDI = 97.7/100.1/98.3 (NS). McCarthy: GCI = (NS) Reynell Developmental Language Scales: Verbal comprehension Scale = 0.2/1.1/0.4 group 2 > 1,3 (S) Expressive Language Scale = −0.3/0.2/–0.1 (NS)	No significant differences in Bayley mental and psychomotor development index and in global cognitive index in McCarthy test between groups. Newborns exposed to TCA had a significant higher score at verbal comprehension scale but within the normal range. Mothers’ depression is associated with less cognitive and language achievement by their children.
Mattson et al. 1999 Prospective cohort study Scale used and severity not specified.	66 exposed to Fluoxetine; 30 control	Not reported	Between 48 and 72 months	Not reported	Fluoxetine	Weschsler Preschool and Primary Scale of intelligence (no results showed, only abstract)	No significant differences in WPPSI-R.
Galbally et al. 2011, Australia Prospective case–control study BDI-II: 16.71/7.85	19 exposed to antidepressant; 22 control	19 exposed to antidepressant; 22 control	Between 18 and 35 months	At any point across the three trimesters of pregnancy	Sertraline, venlafaxine, fluoxetine, fluvoxamine, citalopram, escitalopram, mianserin, mirtazapine, paroxetine	Bayley: Cognitive = 13.5/13.16 (NS), Communication: Receptive = 12/11.42 (NS); Expressive = 11.24/10.53 (NS); Motor: fine motor = 14.18/12.84 (NS); gross motor = 14.18/12.89 (NS); Socio-emotional scaled score = 11.06/10.61 (NS).	No significant differences between groups. They found only a differences on the motor subscale with a moderate Cohen’s effect size *d* = 0.47 for fine motor and *d* = 0.43 for gross motor (*p* = 0.07 and *I* = 0.09, respectively).
Casper et al. 2003, USA Follow-up study Structured Clinical Interview for DSM-IV (SCID-I) Depression rating (Likert scale 1–10):4.8/6.1 Beck Depression Inventory (BDI): 21.3/24.0	31 exposed to SSRI; 13 depressed untreated	31 exposed to SSRI; 13 exposed to untreated depression	At birth, between 6 and 40 months	1st, 2nd, 3rd trimester	SSRI (paroxetine, fluoxetine, sertraline, fluvoxamine) and unspecified psychotherapy for both groups.	Bayley: MDI = 91/94 (NS), PDI = 90/98.2 (S), BRS = 76/89.5 (NS after correction for APGAR score). Motor quality and fine motor movement = (S)	No significant differences in Bayley mental development scores between groups. Significant lower scores for psychomotor development index and motor quality in the exposed group. The behavioral rating scale is not significant only after correction for Apgar score that is lower in exposed newborns.
Nulman et al. 2012, Canada Prospective cohort study Visual analogue scale (0–10): 2.46/3.54/4.55/0	62 exposed to SNRI; 62 exposed to SSRI; 54 depressed untreated; 62 control	62 exposed to SNRI; 62 exposed to SSRI; 54 exposed to untreated depression; 62 control	Between 3 years and 6 years, 11 months	81 exposed throughout pregnancy, 21 1st trimester only, 4 1st and 2nd, 2 2nd only, 11 2nd and 3rd, 5 in the 3rd only	Venlafaxine/sertraline paroxetine, citalopram, fluoxetine, fluvoxamine.	Wechsler Preschool and Primary Scale of Intelligence 3rd Ed.: Full scale IQ = 105/105/108/112. SNRI and SSRI group < control (S), Verbal IQ = 106/107/109/113. SNRI and SSRI group < control (S), Performance IQ = 103/102/105/108. SSRI < control (S)	Babies exposed to SNRI or SSRI had significant lower IQ than control, but there was no significant difference for the baby of untreated depressed mothers. Significant predictors for the child’s IQ were maternal IQ and child’s sex. None result was predicted by dose or duration of antidepressant treatment in pregnancy.

### Short-term effects

#### Antidepressants

The five studies that met inclusion criteria found significant differences in the neurobehavioral scores on both the NBAS and NNNS of newborns exposed to drugs, those exposed to untreated maternal depression, and newborns of healthy women (Smith et al. 2013; Salisbury et al. 2011; Rampono et al. 2009, Ferreira et al. 2007; Zeskind and Stephens 2004); only one study did not find any significant differences (Suri et al. 2011). The most common finding was that infants exposed to drugs displayed more tremors, agitation, irritability, spasms, and hyper or hypotonia than infants of healthy women. Some of these symptoms have been referred to as “neonatal adaptation syndrome” (Grigoriadis et al. 2013), although the studies described here tend to present a wider range of motor and behavioral symptoms, and all studies have used standardized tests of neurodevelopmental outcomes administered by trained staff, as opposed to questionnaire or clinical notes.

In a recent study, Smith et al. (2013) compared neurobehavioral outcomes between neonates who were born to euthymic women who either took or did not take an SSRI during the last trimester of pregnancy. The study found significant differences on motor cluster scores on the NBAS. Exposed infants had lower scores than unexposed, specifically on the pull-to-sit item on the motor cluster, which measures traction, head control, and neck muscle strength. The significant differences on motor cluster became marginal (*p* = 0.05) after adjustment for gestational age. No other significant differences were found regarding infants sleep state and motor activity. Since other studies have documented that exposure to maternal depression during the third trimester could affect the newborn, only women who were euthymic during the third trimester were used as the comparison group (Hernandez-Reif et al. 2006; Hollins 2007; Lundy et al. 1999b). However the study of Smith et al. (2013) was very limited due to the number (five) of exposed infants assessed.

Salisbury et al. (2011) found lower scores on quality of movement, such as hypertonic reflexes, startles, tremors, back-arching, and a greater number of the central nervous system stress signs, in infants exposed to SSRI compared with those exposed to untreated depression or to infants of healthy women. These differences were not related to depression severity or timing or length of SSRI exposure. Newborns exposed to depression had the highest quality of movement scores but significantly lower scores on attention items compared to the other two groups. These findings are similar to other studies cited above, even though they used a more recent test, the NNNS test, developed by Lester and Tronick (2004). The NNNS test assesses the following: neurological functions, as active and passive tone, reflexes and central nervous system integrity; behavioral items including state, sensory, and interactive response; stress and abstinence items. In the other three selected studies, different antidepressants that work on reuptake of norepinephrine or dopamine, such as venlafaxine and bupropion, were used, in addition to SSRI. Main effects were found on the motor and autonomic clusters of the NBAS, which were similar to other studies cited above (Rampono et al. 2009; Ferreira et al. 2007; Zeskind and Stephens 2004).

Suri et al. (2011) reported no significant differences between newborns of mothers with a history of major depressive disorder, those exposed to antidepressants in utero, and a control group born to healthy mothers. The newborns exposed to antidepressant had significantly lower scores on the rapidity of buildup item under the range of state cluster and on the response to inanimate auditory stimulation item under the orientation cluster and higher score on the defensive scale under the motor cluster when compared to the other two groups. The differences were however small and could be due to some of the limitations of the study: the numbers in each group were small, the grouping of multiple antidepressants did not allow for the differentiation of the effects of each single drug, and the use of different settings at home or in hospital for the NBAS assessment.

#### Alternative therapies

Only two studies on alternative therapies for maternal depression and the short-term effects on infant neurodevelopmental outcomes met our inclusion criteria. Field et al. widely studied the effects of depression on the health of women and babies during pregnancy and the benefits of massage therapy for pregnant women (Field 2011; Field et al. 2006; Field et al. 1999). In two randomized clinical trials (Field et al. 2004, 2009), they assessed the effects of massage therapy. The NBAS was used to examine the neurobehavioral development of infants in both studies. The massage therapy was carried out over two sessions of 20 min per week during the second and third trimester of pregnancy. The massage was performed by “significant others” of the women who were then trained by massage therapists.

In the 2004 study, newborns born to mothers in the massage therapy group had better performance on the NBAS with significant better scores than the control group on the habituation, range of state, autonomic stability, withdrawal scales, depressed scale, and motor maturity scale. In the study conducted in 2009, the newborns of the massage group performed significantly better on the NBAS on habituation, orientation, motor, and depression score items. In addition, they had lower cortisol levels that could be related to the lower cortisol level of massage therapy mothers. Moreover, after the massage therapy, the newborns tended to have better neonatal outcome with fewer incidences of low birth weight and prematurity in both studies.

### Long-term effects

To evaluate the long-term effects of drugs or alternative therapies, babies’ outcomes were divided into three categories: psychomotor, mental, and cognitive development, according to the tests used in the selected papers.

#### Antidepressants

Four studies (Hanley et al. 2013; Casper et al. 2011; Mortensen et al. 2003; Casper et al. 2003) found significant negative associations between treatments with antidepressants and psychomotor development scores on the Bayley scale (Bayley 1993) and on the Boel test (Junker et al. 1982). In a recent study, Hanley et al. (2013) studied the effects of exposure to SSRI on 10-month-old babies whose mothers took antidepressants throughout the pregnancy. They found that infants who were exposed to drugs in the antenatal period scored significantly lower on the gross motor, social-emotional, and adaptive behavior items compared with those not exposed. They also investigated the effects of maternal mood during pregnancy and postpartum on infants’ development, and found no significant effects of exposure to antidepressants in any of Bayley items.

Similar results were found by Mortensen et al. (2003). A higher percentage of abnormal Boel items, a psychomotor development test based on 14 items (Junker et al. 1982), were found among exposed compared those who were not exposed to antidepressants and other psychiatric drugs. Casper et al. conducted two studies (2003, 2011) to evaluate the effects of antenatal exposure to antidepressants on children born to depressed mothers. In the first study, they found significant lower scores for the Psychomotor Development Index and Motor Quality Factor of the Bayley Behavioural Rating Scale (a subscale of the Bayley that assesses qualitative aspects of the child’s behavior during the testing situation) in the group exposed to antidepressants compared with unexposed children. In the last study, they also evaluated the effects of the duration of exposure to SSRI and found a significant negative correlation between the length of the exposure and the psychomotor development index, while no significant correlations were found with the mental development index. Oberlander et al. (2004) did not found any significant differences in the Bayley assessment at 2 and 8 months in the Psychomotor Development Index (PDI) between newborns exposed or not exposed to antidepressants. They found a higher incidence of poor neonatal adaptation syndrome at birth in newborns exposed to SSRI alone or in combination with benzodiazepine, especially those who were exposed to the combination of paroxetine and clonazepam. Within the entire exposed group, 30 % of newborns, compared with 9 % of control group, showed symptoms of mild respiratory distress, hypotonia, jittery, tremors and hypertonia, cardiac arrhythmia, bradycardia, and hypoglycemia. After 48 h, symptoms had disappeared in all babies. These findings are in agreement with a recent study by Jordan et al. (2008), and a review by Moses-Kolko et al. (2005) where poor neonatal adaption syndrome, including SSRI withdrawal, SSRI toxicity, serotonergic excess, serotonergic CNS adverse effects, serotonin syndrome, and neonatal behavioral syndrome, affects 25–30 % of newborns whose mothers took SSRI antidepressants during pregnancy, with an overall risk ratio of 3.0 (95 % CI 2.0–4.4).

None of the selected studies found significant differences in the Mental Development Index (MDI) of the Bayley scale or in the General Cognitive Index (GCI) of the McCarthy scale (Table 2). However significant differences were found in the Wechsler Preschool and Primary Scale of Intelligence (Nulman et al. 2012). In this recent study, Nulman et al. (Nulman et al. 2012) compared the IQ of children exposed to antidepressants during pregnancy with the IQ of children of mothers with untreated depression and the children of healthy mothers. They found that children exposed to venlafaxine or SSRI had a significantly lower score in the full-scale IQ test, in the verbal IQ scale, and in the performance IQ scale of the WPPSI compared to the control group. The IQ of children whose mothers had untreated depression was similar to those of children exposed to drugs, but the IQ value was not significantly lower compared with the control group. Neither the length of the exposure during the pregnancy nor the dosage of the drugs had an effect on any cognitive or behavioral outcome. In her previous studies Nulman et al. (Nulman and Koren 1996, Nulman et al. 1997, Nulman et al. 2002) had established that exposure to tricyclic antidepressants and fluoxetine do not adversely affect the cognitive, language, and behavioral development of children, as measured by the MDI of the Bayley, the McCarthy, and the Reynell scales. The duration of depression and the number of episodes after delivery were negatively associated with IQ and language development.

Other authors (Galbally et al. 2011; Reebye et al. 2002) did not find any significant differences in the cognitive and language scores, as measured by the Bayley, of children exposed to antidepressants and those in the control group. Galbally found a difference on the motor subscale with a moderate Cohen’s effect size for fine and gross motor. Neither Mattson et al. (1999) with the WPPSI nor Morison et al. (2001) with the Bayley found any significant differences between children exposed to SSRI and control group.

#### Alternative therapies

Only one study on the therapeutic effects of dietary DHA by Makrides et al. (2010) met the criteria to be included in the review. The therapeutic effects of omega-3 during pregnancy were shown to improve gestational outcomes, and its therapeutic use in treating depression has been confirmed by many authors (Larqué et al. 2012; Su et al. 2008), and interestingly, this is the only randomized controlled trial of this systematic review.

Makrides et al. (2010) evaluated the neurodevelopmental effect of DHA treatment on young children, assessed at 18 months. They did not find any significant differences in the cognitive, language, motor, social-emotional, and adaptive behavior standardized scores on the Bayley Scale III Edition (Bayley 2005), between children exposed to DHA-rich fish oil capsules and vegetable oil capsules during pregnancy and a control group of children exposed to placebo. Although in secondary analyses, they noticed two apparently contrasting effects: fewer children in the DHA group had delayed cognitive development compared with the control group, and girls exposed to higher-dose DHA in utero had lower language scores and were more likely to have delayed language development than girls from the control group. In terms of maternal depression, the DHA capsules were not better than control treatment to treat or prevent postpartum depression. The main significant effects found in this study were a decrease in preterm birth (<34-week gestation) and low birth weight in the DHA group compared with the control group.

## Discussion

This review summarizes the current literature on the neurodevelopmental outcome of children whose mothers were treated for antenatal depression with antidepressants or alternative therapies. Up until now, the majority of the literature only studied the effects of antidepressants; only a few studies have investigated the effects of alternative therapies on infants. The evidence suggests that antidepressants lead to short-term adverse effects on newborns compared with a control group. The motor and autonomic systems are most affected. Studies show that babies who are exposed to drugs present with more tremors, agitation, irritability, spasms, and hypertonia or hypotonia. Many symptoms are however transient and mild (Jordan et al. 2008; Moses-Kolko et al. 2005). The symptoms found in the newborns could be due to toxicity of the SSRI drugs or to the withdrawal syndrome; a single explanation has still not been found. However, none of these studies were randomized controlled trials, and therefore, depression per se may account for at least some of the effects attributed to antidepressants.

In the long-term, some studies have shown a significant effect of antidepressants on psychomotor development, with delay on motor development on the Bayley and Boel assessments. However, other authors have found no significant difference between those who are exposed and the control group. No differences have been found for mental development of the infant: only Nulman et al. (2012) found significantly lower IQ in children exposed to drugs compared with the control group, on the WPPSI. However, the values were similar in children whose mothers were depressed and treated when compared with children whose mothers were depressed but untreated. They also evaluated the effects of the length of exposure and dosage of the drugs and did not find any significant difference. Moreover, the duration of depression and the number of episodes after delivery were negatively associated with IQ and language development, respectively. Again, this suggests that at least some of the neurodevelopmental differences identified in this kind of studies are due, at least in part, to depression per se. Indeed, there is strong evidence that depression per se can adversely affect the neurodevelopment and emotional development of the offspring through in utero biological programming of fetus development and of maternal caregiving behavior (Glover 2011; Pawlby et al. 2011). The potential rescuing effects of antidepressants on some of these biological mechanisms, such as hypercortisolemia (and Pariante and Lightman 2008), could explain why antidepressant treatment in our review is associated with only minor developmental abnormalities. Of note in this regard is the fact that for obstetric complications, more severe forms of depression (for example, diagnosed as cases using clinical interview as opposed to symptoms scale) tend to have more severe complications (Grote et al. 2010). However, the studies described in this report, which often lack information on diagnostic criteria or symptoms severity, do not allow us to draw any conclusion on the impact of these factors on neurodevelopmental outcomes, and indeed, epidemiological studies have shown that even milder form of depression and anxiety can affect long-term offspring behavior (Glover 2011).

The alternative therapies analyzed in this review did not have any negative impact on children in the short- or long-term and found some positive effects. Massage therapy led to better NBAS scores than control group, and in the DHA therapy group, there were fewer children with delay on cognitive development even if therapy was not significant better than placebo to treat depression. It is important to underline the absence of studies about alternative therapies that look at the impact on the newborn. While overall, these studies show important results with this kind of treatment, the number of studies is small and more randomized/controlled research is needed in this field.

## Limitations

As mentioned above, it is important to consider factors other than treatments for maternal depression that could influence neurodevelopmental outcomes of newborns and children. First, the current mental state of the mothers was not considered in all studies. In some cases, mothers only had a history of depression and were taking medication only as prevention or maintenance treatment. The adverse effect of maternal depression on the newborn is well known (Grote et al. 2010). It is important therefore to include the mother’s current mental state when examining the contribution of treatment in altering the neurodevelopmental outcome of the babies. Another limitation is the timing and the length of exposure to the treatment, especially drugs. Casper et al. (2003, 2011) was the only study that considered different trimesters of exposure to antidepressants, and he found a negative correlation between the length of the exposure and the behavioral rating scale on the Bayley assessment. Finally, it is important again to stress that only the studies on alternative therapies (massage therapy and omega-3 fatty acid) were randomized controlled trials with placebo groups; the other studies were prospective or retrospective studies.

## Conclusions

To our knowledge, this is the first review that has examined the neurodevelopment outcomes of babies exposed in utero to antidepressants and to alternative therapies. While the short-term effects seems to be more consistent, these tend to be mild and self-limiting; in contrast, the results of studies examining long-term effects are more varied, and it is virtually impossible to disentangle the effects of antidepressants from the effects of depression per se. Finally, our findings suggest that alternative therapies may be safer options. Future studies are necessary to test new alternative therapies other than drugs and to provide controlled data on the effects of medications in pregnancy on the neurodevelopmental outcomes of children.
